# Curriculum Innovations: Inspiring Neurology Residents to Pursue Fellowship Training in Movement Disorders

**DOI:** 10.1212/NE9.0000000000200074

**Published:** 2023-05-10

**Authors:** Emily Butler, Jack Slatton, Sheffield Sharp, Lauren Schattinger, Maxim Turchan, Phillip D. Charles, Kelly A. Harper

**Affiliations:** From the Department of Neurology (E.B., S.S., L.S., M.T., P.D.C., K.A.H.), Vanderbilt University Medical Center, Nashville, TN; and Marnix E. Heersink School of Medicine (J.S.), University of Alabama, Birmingham.

## Abstract

**Introduction and Problem Statement:**

The aging US population has led to the increased prevalence of neurodegenerative diseases and the critical need for specialists with advanced training in the management of these conditions. Focus on Common Movement Disorders (FOCMD), a 2-day educational course hosted by Vanderbilt University, was started 16 years ago to provide neurology residents with exposure to the diagnosis and treatment of movement disorders.

**Objectives:**

The aim of the course was to provide early-career neurology residents with relevant exposure to the field of movement disorders, through which we hope to increase medical knowledge of movement disorders and common Food and Drug Administration (FDA)-approved therapies and inspire residents to pursue fellowship training in the field.

**Methods and Curriculum Description:**

FOCMD consists of lectures and small-group workshops that provide an overview of common movement disorders and approved therapies. All North American neurology residency program directors are invited to nominate a first-year or second-year resident. Attendees are administered standardized multiple-choice precourse and postcourse examinations to assess foundational knowledge of common movement disorders and FDA-approved therapies and to measure acquisition of the course material. Past participants are regularly surveyed to gauge their impression of the course's effect on fellowship selection and their utilization of therapies common in the treatment of movement disorders.

**Results and Assessment Data:**

Since 2008, FOCMD has trained 854 neurology residents from 113 programs. Between 2010 and 2020, 507 residents completed the precourse and postcourse examinations. There was an increase of 22.4 (95% CI 20.67–24.49; *p* < 0.001) percentage points between the precourse and postcourse examinations or an additional 3.8 questions were answered correctly. Follow-up surveys were sent to 414 past participants, and 116 were completed. Survey responses revealed that 84% of past attendees completed a fellowship, 44% of which were in the field of movement disorders. In addition, 82% of past participants reported that the course affected subspecialty selection and 63% reported treating patients with movement disorders in their current practice.

**Discussion and Lessons Learned:**

Experience with FOCMD has shown it to be successful in introducing neurology residents to the subspecialty early in their career and increasing medical knowledge on movement disorders. An educational program of this format may also positively influence fellowship selection.

## Introduction and Problem Statement

Americans 65 years and older make up 16% of the total population, and that number is expected to increase to 23% by 2060.^1^ As the US population ages, the prevalence of neurodegenerative diseases, specifically movement disorders, is also increasing. An estimated 42 million Americans have been diagnosed with a movement disorder, and as the population ages, it is inevitable that the burden of disease will continue to increase proportionally.^2^ Now, more than ever, there is a need for subspecialty physicians who are trained in the diagnosis and management of these conditions.

Nationwide, there is a critical shortage of neurologists to meet the growing needs of the population. By 2025, the United States is predicted to need 19% more neurologists than are currently available.^3^ Yet each year only 2.8% of graduating medical students elect to pursue residencies in neurology,^4^ and of the neurologists graduating from residency programs, only 11.3% choose fellowship training in movement disorders.^5^ This is compared with vascular neurology and stroke, where 14.0% of new neurologists choose to pursue fellowship training.^5^ In 2020, 25% of available movement disorder fellowship positions went unfilled.^6^ Difficulties in recruiting trainees to the field of movement disorders is 2-fold. First, neurology residency curriculum requirements do not dictate dedicated rotations in movement disorders, limiting subspecialty exposure to early-career residents. Moreover, many residency programs do not have sufficient movement disorders faculty to train residents, further restricting exposure to the field. A 2017 survey reported that 46% of residents in adult neurology programs felt that they did not have enough exposure to make a fellowship decision.^7^ Providing residents with educational resources and exposure to the field of movement disorders early in their training may help compensate for the limited exposure provided by their program.

The Department of Neurology at Vanderbilt University developed Focus on Common Movement Disorders (FOCMD) to fill the educational gap in neurology residency curricula and to foster interest in the field of movement disorders. Over the past 16 years, FOCMD has offered first-year and second-year adult neurology residents a 2-day, educational course that surveys the diagnosis and treatment of common movement disorders, including Parkinson disease, spasticity, dystonia, and tremor.

## Objectives

The aim of the course was to provide early-career neurology residents with relevant exposure to the field of movement disorders through which we seek to achieve the following 2 outcomes: (1) to increase residents' medical knowledge of movement disorders and common Food and Drug Administration (FDA)-approved therapies and (2) to inspire them to pursue fellowship training in movement disorders.

## Methods and Curriculum Description

The FOCMD curriculum was developed by senior-level experts in the field of movement disorders to provide a broad overview of common movement disorders. FOCMD is a 2-day, in-person course consisting of both didactic lectures and hands-on, small-group workshops that provide a broad introduction to FDA-approved therapies for movement disorders, including neurotoxin injection, deep brain stimulation, and therapies delivered through continuous pumps (Table 1). At the conclusion of the course, attendees should have an introductory understanding of how to accurately diagnose common movement disorders, select patients for FDA-approved therapies, administer neurotoxin injections, program deep brain stimulators and intrathecal baclofen pumps, and medically manage patients with common movement disorders. The course is run by senior movement disorder faculty with expertise in the topics selected.

**Table 1:** FOCMD Curriculum Overview^a^

Lectures	Overview of common movement disorders Neurotoxin injection and intrathecal therapy for spasticity and dystonia Deep brain stimulation for common movement disorders Medical management of common movement disorders
Small-group workshops	Neurotoxin injections Deep brain stimulation Therapies through continuous pump delivery Video case studies

Abbreviation: FOCMD = Focus on Common Movement Disorders.

a Course lectures and workshops have remained the same, except for “Medical Management of Common Movement Disorders,” which was added to the curriculum in 2012.

All North American adult neurology residency program directors are provided with detailed information on the course and are invited to nominate a first-year or second-year resident. The course is limited to 48 participants to foster an open dialog between participants and faculty instructors, particularly in the small-group workshop setting. Preference is first given to residents at a training program without dedicated movement disorders specialists. Then, first-year residents are selected, followed by residents from programs that have not sent participants in recent years, if ever.

Two outcomes were measured through the administration of past participant surveys and standardized, multiple-choice precourse and postcourse examinations. The effect of the course on fellowship selection and the utilization of therapeutics common in the treatment of movement disorders represent the primary outcome while the acquisition of medical knowledge represents the secondary outcome.

### Standard Protocol Approvals, Registrations, and Patient Consents

This project is approved by the Vanderbilt University Institutional Review Board (IRB#212394). No patients were included in this study.

Past participants were emailed links to surveys using Research Electronic Data Capture (REDCap), a secure web-based platform for managing online surveys and databases, to investigate the effect of the course on their career decisions. Surveys were distributed in May 2017 and May 2020 to participants who would have already completed fellowship training based on their year of attendance. The survey consisted of 6 questions and took approximately 5 minutes to complete (eAppendix 1, links.lww.com/NE9/A25). The automated reminders function of REDCap was used to send reminders to past participants to complete the survey. Participants were asked whether they pursued advanced fellowship training and, if so, in what subspecialty. They were also asked whether FOCMD influenced subspecialty selection, whether their practice included patients with movement disorders, and what, if any, common FDA-approved therapies they use—neurotoxin injection, deep brain stimulation, intrathecal baclofen, levodopa suspension through pump, apomorphine, and/or medications for Parkinson-associated psychosis.

Residents completed standardized examinations consisting of 17 multiple-choice questions developed by movement disorders experts before the course to assess their foundational knowledge of common movement disorders and FDA-approved treatments. To quantify the acquisition of educational material, participants were administered a second, identical examination at the conclusion of the course.

### Data Availability

The past participant survey is included in the supplemental material. Deidentified survey responses and examination scores not published within this article are available on request.

## Results and Assessment Data

Since 2008, FOCMD has trained 854 neurology residents from 113 programs. Between 2011 and 2020, 122 first-year residents, 368 second-year residents, and 2 third-year residents attended the course. Past participants for whom we had valid email addresses (n = 414) were sent course effect surveys. There were 116 surveys completed and returned for a response rate of 28%. Survey responses revealed that 97 participants (84%) went on to complete a fellowship, and of those who pursued advanced training, 43 participants (44%, 43/97) chose movement disorders. In addition, most of the past participants (82%) reported that the course affected their subspecialty selection, and most (63%) said that their current practice includes treatment of patients with movement disorders.

Survey respondents reported using a variety of FDA-approved therapies for movement disorders in their current practice, regardless of the subspecialty they chose (Table 2). Sixty-three percent (n = 73) of participants reported using neurotoxin injections; 47% (n = 54) reported using deep brain stimulation; 14% (n = 16) reported using intrathecal baclofen; 19% (n = 22) reported using levodopa suspension through pump to treat their patients; 19% (n = 22) reported that they use apomorphine in their current practice; and 72% (n = 83) reported using medications for Parkinson-associated psychosis.

**Table 2:** Use of FDA-Approved Therapies in Current Practice

	All respondents (n = 116), n (%)	Movement disorders fellowship (n = 43), n (%)	No fellowship or non-movement disorders fellowship (n = 73), n (%)
Neurotoxin injections	73 (63)	39 (91)	34 (47)
Deep brain stimulation	54 (47)	38 (88)	16 (22)
Intrathecal baclofen	16 (14)	6 (14)	10 (14)
Levodopa suspension through pump	22 (19)	22 (51)	0 (0)
Apomorphine	22 (19)	18 (42)	5 (7)
Medications for Parkinson-associated psychosis	83 (72)	38 (88)	45 (62)

Abbreviation: FDA = Food and Drug Administration.

Between 2010 and 2020, 507 participants completed the precourse and postcourse examinations and were included in the analysis. Examination scores from the 2013 course were not available because of a clerical recording error. All scores were reported out of 100. The mean pretest score was 53.8 (3.6) and the mean post-test score was 76.4 (3.2), which corresponds to an additional 3.8 (0.4) questions answered correctly. There was an average increase of 22.4 (95% CI 20.67–24.49; *p* < 0.001) percentage points from the precourse to postcourse examination, corresponding to a Cohen *d* of 8.45, a very large effect size.

## Discussion and Lessons Learned

Data from past participant surveys suggest that FOCMD has had a positive influence on the selection of fellowship training in movement disorders among participants. Compared with the national percentage of neurology residents choosing fellowship training in movement disorders (11.3%), course participants completed movement disorders fellowship training at a frequency of approximately 4 times higher (44%). This suggests that an educational program of this nature directed at junior residents could be useful in calling attention to and encouraging interest in under-represented subspecialties.

A large proportion of past participants reported treating patients with movement disorders and using common therapeutic options in their current practices. Even if these participants elected not to pursue a fellowship in movement disorders, these responses highlight the relevance of the course material. Moreover, compared with pre-examination scores, postexamination scores were consistently improved following the FOCMD course, indicating elevated awareness and medical knowledge concerning common movement disorders. Overall, these data indicate that the course provides impactful exposure to early-career residents.

There are several limitations that must be considered when interpreting these results. Selection bias among the nominations by residency program directors could skew the data toward residents with existing interest in movement disorders. In addition, asking past participants whether FOCMD influenced their subspecialty selection introduces ambiguity. It would not be appropriate to conclude that FOCMD uniquely influenced subspecialty selection and does not completely capture someone's decision to pursue fellowship training. Many past participants did not retain active institutional email addresses after completing residency and were unable to be contacted to complete the survey. Of those who received the survey, response bias to past participant surveys could exaggerate positive trends in the course effect. Another potential limitation is that, while the precourse and postcourse multiple-choice examination questions were developed by movement disorders experts, validity arguments and data were not gathered before implementation of the examination. In addition, we did not collect demographic information on sex, whether nominees were historically under-represented in medicine, or whether their residency training program had movement disorders neurologists.

With these limitations in mind, the FOMCD course experience provides helpful lessons on the implementation of an educational course of this format. Because institutional emails expire after an individual's affiliation ends, we now collect personal email addresses in an effort to maintain long-term contact. Going forward, we plan to continue surveying past participants to collect longitudinal data on their training and current practices. Based on the experience with FOMCD, Vanderbilt University launched the Junior Faculty Forum on Movement Disorders in 2019 for instructors and assistant professors at academic centers within 7 years of the completion of fellowship training. Junior faculty have a need for advanced training in movement disorders to remain abreast of the latest technology and therapeutic options available to patients. Participants must be nominated by their department chair or division director. The Junior Faculty Forum follows a similar course format to FOCMD, with most educational activities occurring in small, hands-on workshops. A unique feature of the Junior Faculty Forum is that each participant is assigned 2 teaching topics. Senior faculty are present only as facilitators because the entire Forum is taught by the participants.

Early exposure to subspecialties of neurology represents a major barrier to fellowship selection, but focused educational programs seem to be beneficial and impactful. Several subspecialty professional societies within the field of neurology, including movement disorders, headache, and epilepsy, have adopted their own courses geared toward first-year and second-year residents to address unmet educational gaps.^8^ In 2016, the International Movement Disorders and Parkinson's Society launched their 2-day, in-person course modeled after Vanderbilt's FOCMD.^9^ For the past 7 years, the Movement Disorders School for Neurology Residents has been successfully providing early-career residents with an overview of common movement disorders, advances in research, and potential career paths within the subspecialty.^9^

FOCMD is an impactful educational program that represents a promising step toward inspiring interest in movement disorders fellowship training. Moreover, experience with this course suggests that early exposure to under-represented subspecialties and the adoption of similar courses using this format could help address future physician shortages.

## Appendix Authors

**Table:**

Name	Location	Contribution
Emily Butler, BA	Department of Neurology, Vanderbilt University Medical Center, Nashville, TN	Drafting/revision of the manuscript for content, including medical writing for content; analysis or interpretation of data
Jack Slatton, BA	Marnix E. Heersink School of Medicine, University of Alabama, Birmingham	Drafting/revision of the manuscript for content, including medical writing for content; analysis or interpretation of data
Sheffield Sharp, BS	Department of Neurology, Vanderbilt University Medical Center, Nashville, TN	Drafting/revision of the manuscript for content, including medical writing for content; analysis or interpretation of data
Lauren Schattinger, BS	Department of Neurology, Vanderbilt University Medical Center, Nashville, TN	Drafting/revision of the manuscript for content, including medical writing for content; major role in the acquisition of data; study concept or design; analysis or interpretation of data
Maxim Turchan, MS	Department of Neurology, Vanderbilt University Medical Center, Nashville, TN	Major role in the acquisition of data; study concept or design; analysis or interpretation of data
Phillip David Charles, MD	Department of Neurology, Vanderbilt University Medical Center, Nashville, TN	Drafting/revision of the manuscript for content, including medical writing for content; major role in the acquisition of data; study concept or design; analysis or interpretation of data
Kelly Ann Harper, BPS	Department of Neurology, Vanderbilt University Medical Center, Nashville, TN	Drafting/revision of the manuscript for content, including medical writing for content; major role in the acquisition of data; study concept or design; analysis or interpretation of data

## Glossary

FDA: Food and Drug Administration
FOCMD: Focus on Common Movement Disorders
REDCap: Research Electronic Data Capture
